# Clinical features of dengue-associated myositis in children: a retrospective study

**DOI:** 10.1016/j.jped.2026.101549

**Published:** 2026-05-06

**Authors:** Raquel O. Birne, Marianna A. Neri, Camille A. Rossetti, Mila O. Sena, Laís P.R. Duarte, Luji I. Takenami, Juliana B. Goulardins, Marcos A. Matos

**Affiliations:** a Department of Post-Graduation, Escola Bahiana de Medicina e Saúde Pública, Salvador, BA, Brazil; b Division of Pediatric, Emergency Department, Hospital Santa Izabel, Salvador, BA, Brazil

**Keywords:** Dengue, Pediatrics, Myositis, Infectious myositis, Rhabdomyolysis

## Abstract

**Objectives:**

The presentation of dengue as infectious myositis is considered an atypical and often underrecognized manifestation. This study aimed to characterize the clinical features and outcomes of pediatric patients diagnosed with dengue-associated infectious myositis, to delineate their sociodemographic profiles at the time of diagnosis, and to evaluate their laboratory findings and clinical outcomes.

**Methods:**

The authors conducted a retrospective observational study in the pediatric department of a tertiary care hospital in the state of Bahia, Brazil, including patients up to 14 years who were admitted with clinical features of infectious myositis and laboratory-confirmed dengue infection between January 2022 and July 2024. Sociodemographic characteristics, clinical manifestations, laboratory parameters and outcomes were analyzed.

**Results:**

Data from 176 patients were analyzed. A higher incidence was observed among male patients (68.8%), with most cases occurring during the winter season (56.25%). The mean age was 9.25 ± 3.22 years. Fever was the most frequent symptom (96%), followed by myalgia (89.2%). Calf pain was reported in 69.3% of cases. Regarding creatine phosphokinase (CK) levels, 46% of patients had values between 1000–5000 U/L, while 17% presented with CK > 5000 U/L at admission. None of the patients developed acute kidney injury or died.

**Conclusions:**

Dengue-associated myositis exhibited a benign clinical course in this symptomatic pediatric cohort. However, these findings suggest that clinical and laboratory parameters may support a risk-based clinical approach to patient monitoring and management; prospective studies are needed to validate risk stratification strategies.

## Introduction

Myositis is defined as the inflammation of a muscle, particularly a voluntary muscle, and may have several causes, including infections, autoimmune diseases, genetic alterations, medications, trauma, electrolyte disturbances, endocrinopathies, or it may be idiopathic [1,2].

Myositis associated with infectious agents has been referred to as infectious myositis and can be caused by various microorganisms, including bacteria, viruses, fungi, and parasites [1]. Among these agents, viruses are the most common cause of infectious myositis, leading to myalgia, polymyositis, or associated rhabdomyolysis. Viruses associated with infectious myositis include influenza, SARS-CoV-2, Epstein–Barr virus, cytomegalovirus, arboviruses, among others [3, 4, 5, 6]. The influenza virus is frequently remembered in respiratory outbreaks; however, despite its relevance, the dengue virus is often overlooked as a cause of this condition [1,4].

In dengue-endemic regions, myositis should be considered as a possible manifestation, especially in children, although other viral etiologies remain common and should be considered in the differential diagnosis [7]. According to the Pan American Health Organization (PAHO), about 4 billion people live in dengue-risk areas, mainly in the Americas, Africa, Asia, the Middle East, and the Pacific Islands. In the Americas, dengue is endemic, with Brazil leading in the number of reported cases. Data from PAHO show an increase in the number of dengue cases in Brazil since 202 [8].

Despite the magnitude of the disease, there is a scarcity of studies on dengue-related myositis in pediatrics, with available data coming from case reports [3,4,9,10]. This age group is particularly affected, and the condition may progress to rhabdomyolysis and even acute kidney injury [11]. Clinically, infectious myositis is characterized by muscle pain, which may impair ambulation, highlighting the need for early diagnosis. Recognition is essential to differentiate it from other severe neurological conditions such as encephalitis, acute flaccid paralysis, and Guillain-Barré syndrome [5,10,12,13].

The aim of this study was to describe the clinical and laboratory manifestations, as well as the outcomes, of dengue-associated infectious myositis in children, emphasizing the importance of early detection. Correct diagnosis prevents invasive and costly interventions, which, besides affecting the well-being of the child and family, also generate unnecessary expenses for the healthcare system.

## Methods

**Figure 1:**
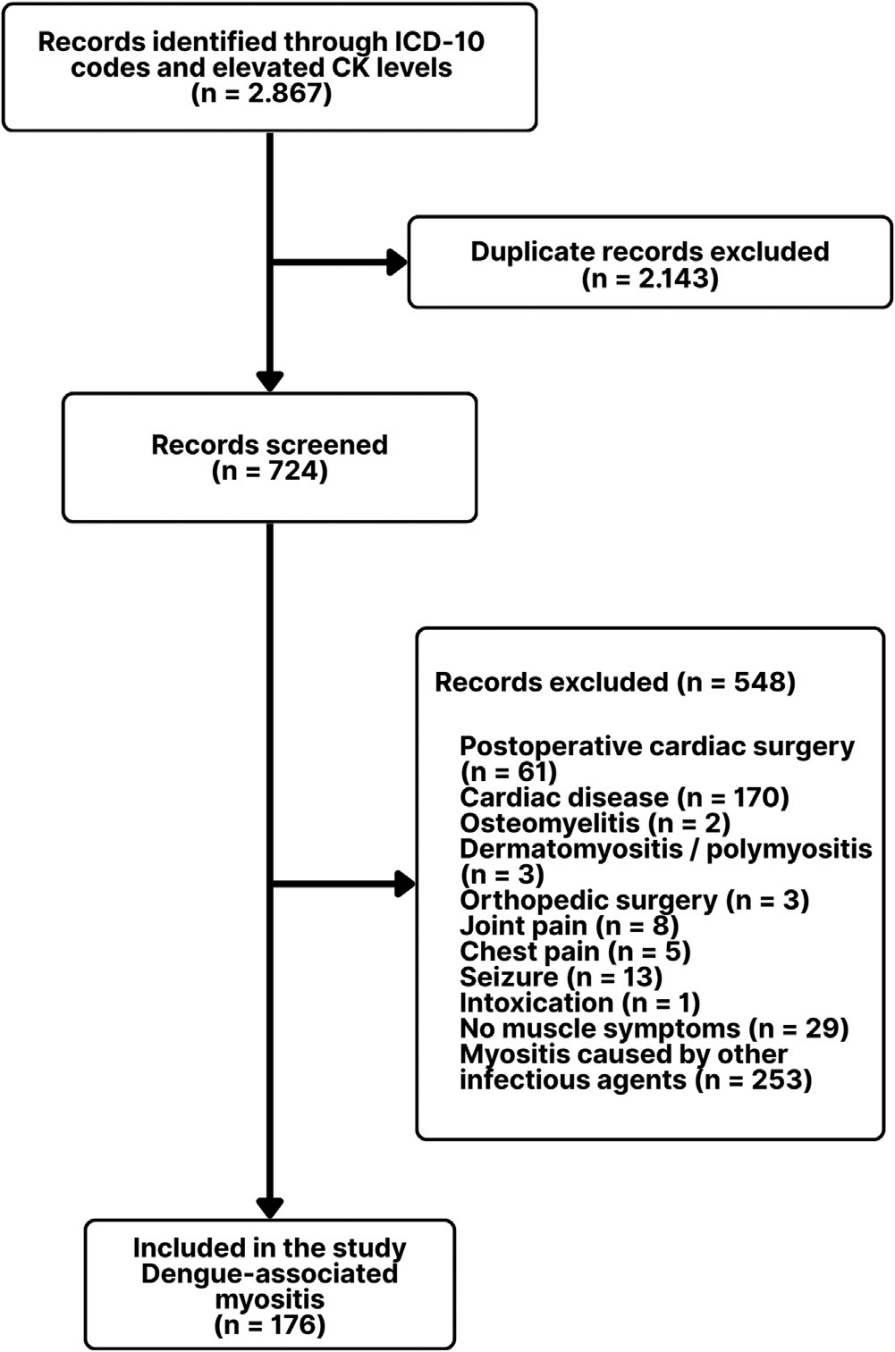
Flowchart of patient selection for dengue-associated infectious myositis.

This was a retrospective, single-center observational study conducted in the pediatric department tertiary hospital in Bahia, Brazil. It included patients up to 14 years of age admitted to the pediatric emergency unit with a diagnosis of dengue-associated infectious myositis between January 2022 and July 2024. Patients discharged after initial care and those admitted to pediatric wards were considered eligible. Identification was through medical records using ICD-10 (International Classification of Diseases, 10th edition) codes related to infectious myositis (M60, M60.0, M60.8, M60.9), myalgia (M79.1), muscle disorders (M62), limb pain (M79.6), and pediatric patients with elevated serum creatine kinase (CK) levels. Patient selection is detailed in Figure 1. Only those with clinical and laboratory evidence of infectious myositis associated with elevated CK levels were included.

This study was designed to characterize patients with suspected infectious myositis whose etiological investigation confirmed dengue infection, rather than to broadly represent patients with a clinical diagnosis of dengue. Therefore, the study population represents a symptomatic subset of patients investigated for muscle involvement rather than an unselected cohort of pediatric dengue cases.

Data were collected using a structured 75 - item form, covering sociodemographic information (age, sex, schooling, admission date), clinical data (symptoms, duration of illness, comorbidities, prior history of myositis, treatment, length of stay, symptom evolution, and outcomes), and laboratory results. Laboratory tests included CK, complete blood count, inflammatory markers, lactate dehydrogenase (LDH), aspartate aminotransferase (AST), alanine aminotransferase (ALT), urea, creatinine, electrolytes, and urinalysis.

Elevated CK was defined as values >192 U/L, with rhabdomyolysis classified as mild (CK 1000–5000 U/L) or severe (CK 5000–10,000 U/L), or when associated with acute kidney injury [14]. Studies in children and adolescents on infectious myositis show variability in the definition of rhabdomyolysis [9,11,15,16]. Therefore, the definitions of mild and severe rhabdomyolysis used in this study were based on thresholds commonly adopted in contemporary pediatric literature on infectious and non-traumatic rhabdomyolysis [14].

Hematological alterations were defined as follows: leukopenia (< 4500 leukocytes/mm³), leukocytosis (> 10,000/mm³), neutropenia (< 1000/mm³), lymphopenia (< 1000/mm³), and thrombocytopenia (< 150,000/mm³) [4]. The neutrophil-to-lymphocyte ratio (NLR) was also calculated and considered altered if ≥ 5.0 [17]. Acute kidney injury was defined according to KDIGO (*Kidney Disease: Improving Global Outcomes*) guidelines [15].

The diagnosis of dengue followed World Health Organization (WHO) criteria, confirmed by either rapid IgM (immunoglobulin M) or NS1 (nonstructural protein 1) tests, both performed by serum immunochromatography, according to symptom duration: NS1 up to the 5th day, and IgM rapid test from the 6th day onward [18, 19, 20]. Laboratory-confirmed dengue infection was defined as a positive NS1 or IgM result in patients presenting compatible clinical features. RT-PCR testing and viral serotyping were not routinely available at the institution and are typically reserved for epidemiological surveillance purposes within the public health system. During patient selection, those with evidence of alternative infectious etiologies associated with muscle symptoms (e.g., influenza) were excluded to reduce diagnostic uncertainty, as illustrated in Figure 1. Laboratory confirmation was interpreted in conjunction with clinical compatibility and exclusion criteria to minimize incidental dengue attribution in this endemic setting.

Data were analyzed using SPSS Statistics software. Descriptive statistics were applied: continuous variables were summarized as means and standard deviations, while categorical variables were presented as frequencies and percentages. Comparative tests were selected according to variable type (categorical or continuous), distribution, and sample size. The Chi-square test (or Fisher’s exact test) was applied for categorical variables; Student’s *t*-test was used for continuous variables; correlations were assessed using Spearman’s test. All statistical analyses were considered significant when *p* < 0.05.

The study was initiated only after approval was obtained from the hospital’s Research Ethics Committee (Approval No 6.915.795).

## Results

### Epidemiological data

Data from the medical records of 176 patients aged 0–14 years with laboratory-confirmed dengue infection were analyzed. The etiological agent was identified by NS1 antigen testing in 139 patients and by dengue IgM testing in 37 patients; 17 patients had both NS1 and IgM positive results. Laboratory confirmation followed WHO diagnostic recommendations according to symptom duration. As dengue is endemic in Brazil, viral isolation and RT-PCR testing with dengue virus serotype determination are not routinely performed and are generally reserved for epidemiological surveillance purposes.

Epidemiological and sociodemographic data are summarized in Table 1. A higher proportion of cases was observed among male patients, and most cases occurred during the winter season.

**Table 1:** Epidemiological data and clinical manifestations.

**Variables**	**n/ %**
**Dengue Infectious Myositis Cases, n**	176
**Sex, *n* (%)**	
Male	121 (68.8 %)
Female	55 (31.2 %)
**Age, years**	
Mean **±** SD	9,25 (± 3.22)
**Previous history of myositis, *n* (%)**	6 (3.4 %)
**Presence of comorbidities, *n* (%)**	18 (10.2 %)
Asthma	7 (4 %)
Asthma and allergic rhinitis (same patient)	1
Obesity and HTLV infection (not specified as I or II)	1
Down syndrome and hypothyroidism (same patients)	1
Autism Spectrum Disorder	4 (2 %)
Mild/asymptomatic aortic insufficiency	1
Epilepsy	2 (1 %)
Idiopathic leukopenia	1
**Season of admission, *n* (%)**	
Summer (December - February)	14 (7.95 %)
Autumn (March - May)	47 (26.7 %)
Winter (June - August)	99 (56.25 %)
Spring (September - November)	16 (9.09 %)

### Clinical manifestations and outcomes

Patients with dengue-associated infectious myositis presented viral prodromal symptoms (fever, respiratory, and gastrointestinal) in addition to muscle complaints, as shown in Table 2. Rash/exanthema, a clinical manifestation commonly seen in dengue infection, was reported in 12.5 % of patients.

**Table 2:** Clinical manifestations and outcomes.

**Variables**	**n/ %**
Prodromal **symptoms, *n* (%)**	
Fever	169 (96 %)
Cough	23 (13.1 %)
Coryza	12 (6.8 %)
Sore throat	7 (4 %)
Vomiting	39 (22.2 %)
Nausea	29 (16.5 %)
Abdominal pain	35 (19.9 %)
Diarrhea	19 (10.8 %)
Rash	22 (12.5 %)
**Muscle signs and symptoms, *n* (%)**	
Myalgia	157 (89.2 %)
Calf pain	122 (69.3 %)
Difficulty walking	71 (40.3 %)
Muscle weakness	10 (5.7 %)
**Type of Hydration Chosen for Treatment, *n* (%)**	
Oral Hydration	52 (29.5 %)
Intravenous Hydration	124 (70.5 %)
**Inpatient Unit Chosen for Treatment, *n* (%)**	
Outpatient treatment after emergency room care	52 (29.5 %)
Admission to the pediatric ward	124 (70.5 %)
**Time to symptom onset (mean ± standard deviation)**	3.7 (±1.6)
**Time to muscle symptom improvement (mean ± standard deviation)**	6.1 (±2.87)
**Length of hospital stay (mean ± standard deviation)**	3.4 (±1.46)

As for treatment, 124 patients (70.5 %) were admitted to a general pediatric ward, where they received intravenous hydration and analgesia.

### Laboratory findings

With respect to laboratory results, leukopenia and thrombocytopenia were frequent findings, as described in Table 3.

**Table 3:** Laboratory tests and clinical outcomes.

**Variable**	**n/ %**
**Dengue Infectious Myositis Cases, n**	176
**CK Values at Admission, *n* (%)**	
192–1000 U/L (no rhabdomyolysis)	65 (36.9 %)
1000–5000 U/L (mild rhabdomyolysis)	81 (46.0 %)
>5000 U/L (severe rhabdomyolysis)	30 (17.0 %)
**CK Values at Admission, mean ± SD**	
192–1000 U/L (no rhabdomyolysis)	557.72 (± 217.48)
1000–5000 U/L (mild rhabdomyolysis)	2579.19 (± 1173.85)
>5000 U/L (severe rhabdomyolysis)	8820.30 (± 4584.53)
**CK at admission by treatment unit, mean ± SD**	
Outpatient Treatment (discharge from the emergency room)	1207.75 (± 1168.20)
Admission to the Pediatric Ward	3604.61 (± 3887.81)
**Lymphopenia (lymphocytes < 1000/mm^3^)**	50 (28.4 %)
**Thrombocytopenia (platelets < 150,000/mm^3^)**	80 (45.5 %)
**Leukopenia (leukocytes < 4500/mm^3^)**	138 (78.4 %)
**Neutropenia (neutrophils < 1000/mm^3^)**	47 (26.7 %)
**CRP, mean ± SD**	0.42 (± 0.64)
**Hemoglobin, mean ± SD**	13.18 (± 1.12)
**Hematocrit, mean ± SD**	38.90 (± 3.1)
**Urea, mean ± SD**	18.74 (± 5.96)
**Creatinine, mean ± SD**	0.49 (± 0.17)
**Neutrophil/Lymphocyte Ratio (NLR), *n* (%)**	
< 5.0	173 (98.3 %)
≥ 5.0 – 10.0	2 (1.1 %)
≥ 10.0	1 (0.6 %)
**Hematuria (dark urine), *n***	4 (2.3 %)

Almost all patients (173 out of 176) had a neutrophil-to-lymphocyte ratio (NLR) ≤ 5.0; only one patient presented NLR ≥ 10.0. However, none of the patients required admission to the pediatric intensive care unit (PICU), nor were there any deaths. Lower CK levels were associated with a higher frequency of lymphopenia, as shown in Table 4. No statistically significant association was observed between CK subgroups and dengue severity.

**Table 4:** Association of CK levels with symptoms, tests and clinical outcomes.

**Characteristics**	**CK 192–1000U/L**	**Mild Rhabdomyolysis** **CK 1000–5000U/L**	**Severe Rhabdomyolysis** **CK > 5000U/L**	**p**
**Sex, *n* (%)**				
Male	37 (56.9 %)	62 (76.5 %)	21 (71 %)	0.033
Female	28 (43.1 %)	19 (23.5 %)	9 (29 %)	0.033
**Previous history of myositis, *n* (%)**				
	2 (3.1 %)	1 (1.2 %)	3 (9.7 %)	0.076
**Dengue severity, n (%)**				
Dengue without warning signs	55 (84.6 %)	74 (91.4 %)	28 (93.3 %)	0.310
Dengue with warning signs	10 (15.4 %)	7 (8.6 %)	2 (6.7 %)	0.310
Severe dengue	0 (0.0 %)	0 (0.0 %)	0 (0.0 %)	-
**Prodromal symptoms, n (%)**				
Fever	63 (96.9 %)	78 (96.3 %)	28 (93.3 %)	0.697
Cough	12 (18.5 %)	7 (8.6 %)	4 (13.3 %)	0.427
Coryza	5 (7.7 %)	5 (6.2 %)	2 (6.6 %)	0.795
Sore throat	4 (6.2 %)	1 (1.2 %)	2 (6.6 %)	0.360
Vomiting	16 (24.6 %)	21 (25.9 %)	2 (6.6 %)	0.122
Nausea	15 (23.1 %)	12 (14.8 %)	2 (6.6 %)	0.325
Abdominal pain Diarrhea Rash	14 (21.5 %) 4 (6.2 %) 12 (18.5 %)	16 (19.8 %) 13 (16.0 %) 8 (9.9 %)	5 (16.6 %) 2 (6.6 %) 2 (6.6 %)	0.779 0.097 0.409
**Muscle signs and symptoms, *n* (%)**				
Myalgia	55 (84.6 %)	74 (91.4 %)	28 (93.3 %)	0.310
Muscle weakness	6 (9.2 %)	2 (2.5 %)	2 (6.7 %)	0.208
Calf pain	47 (72.3 %)	54 (66.7 %)	21 (70.0 %)	0.761
Difficulty walking	27 (41.5 %)	27 (33.3 %)	17 (56.7 %)	0.082
**Type of Hydration Chosen for Treatment, *n* (%)**				
Oral	32 (49.2 %)	16 (19.8 %)	4 (13.3 %)	< 0.01
Intravenous	33 (50.8 %)	65 (80.2 %)	26 (86.7 %)	< 0.01
**Inpatient Unit Chosen for Treatment, *n* (%)**				
Outpatient treatment after emergency care	34 (52.3 %)	17 (21.0 %)	1 (3.3 %)	< 0.01
Admission to the pediatric ward	31 (47.7 %)	64 (79.0 %)	29 (96.7 %)	< 0.01
**Length of hospital stay, days, mean ± standard deviation**				
	3.90 (±2.12)	3.20 (±1.06)	3.31 (±1.31)	0.087
**Laboratory alterations, *n* (%)**				
Leukopenia	50 (76.9 %)	67 (82.7 %)	21 (70.0 %)	0.187
Neutropenia	18 (27.7 %)	22 (27.2 %)	7 (23.3 %)	0.934
Thrombocytopenia	31 (47.7 %)	38 (46.9 %)	11 (36.7 %)	0.567
Lymphopenia	26 (40.0 %)	19 (23.5 %)	5 (16.7 %)	0.030
NLR ≥ 5.0	2 (3.1 %)	0 (0.0 %)	1 (3.3 %)	0.401
Elevated creatinine	2 (3.1 %)	1 (1.2 %)	0 (0.0 %)	0.274

Although 4 patients (2.3 %) presented with macroscopic hematuria or reported “dark urine” upon admission, none of the patients developed acute kidney injury, required renal replacement therapy, or received bicarbonate therapy.

Regarding CK levels at admission, most patients had values between 1000–5000 U/L. When comparing CK levels with the presence of myalgia, muscle weakness, and calf pain, no direct association was found between higher CK levels and these symptoms.

## Discussion

This study describes the sociodemographic, clinical, and laboratory characteristics, as well as the outcomes, of pediatric patients diagnosed with dengue-associated infectious myositis, in order to provide a better understanding of the condition and to improve clinical care protocols.

Dengue-associated infectious myositis is an underrecognized and underreported atypical manifestation that should be considered in tropical and subtropical countries [1,2,10,20,21]. In the studied cohort, dengue infection was laboratory-confirmed and cases with evidence of alternative infectious etiologies associated with myositis were excluded during chart review to reduce diagnostic uncertainty in an endemic setting. In the study, 176 children with dengue-related myositis were evaluated, representing the largest pediatric case series of dengue myositis reported to date. Two Brazilian studies on infectious myositis in pediatric patients did not report dengue among their cases [22,23], although one of them mentioned dengue as a potential etiologic agent [23].

In the present cohort, dengue-associated myositis was more frequent in males, with a statistically significant difference observed across all CK subgroups and more prevalent during the winter season. The most frequent prodromal symptoms were fever, followed by vomiting and abdominal pain. Rash was reported in 22 patients (12.5 %). Patients presented with respiratory symptoms such as cough (13.1 %) and coryza (6.8 %). Approximately 70 % of patients required hospitalization for intravenous hydration therapy, particularly in cases with moderately or severely elevated CK levels.

Mild (MR) and severe rhabdomyolysis (SR) occurred in 63 %. Both the need for intravenous hydration and the length of hospitalization were significantly higher among patients with severe rhabdomyolysis. Higher CK levels were not directly associated with more severe muscle symptoms; however, lymphopenia was more frequently observed among patients with lower CK levels (Table 4), suggesting that hematological alterations may reflect systemic viral response rather than the severity of muscle injury. The average time for symptom resolution was 6.1 days, and the mean hospital stay was 3.4 days. Hematuria was identified in 2.3 % of cases, but no patient developed acute kidney injury, required dialysis, bicarbonate therapy, or died. Laboratory findings most commonly revealed leukopenia, followed by thrombocytopenia, lymphopenia, and neutropenia.

As in classical cases of infectious myositis, all patients reported muscle complaints upon admission. Calf pain was the most frequent classic symptom, followed by difficulty walking. Muscle-related symptoms were dominated by myalgia, reported in nearly 90 % of patients. Only 5.7 % presented with weakness, but none required further investigation, as more severe causes of muscle weakness were ruled out by physical examination and their benign clinical course.

Although dengue can be a potentially severe disease, patients with myositis did not require neurology or orthopedics consultations, nor PICU admission. Complex tests, such as ultrasound, electromyography, or muscle biopsy, were unnecessary, as patients evolved with a benign course. Treatment followed WHO recommendations and consisted of intravenous hydration with isotonic solutions and analgesia with dipyrone or acetaminophen, while avoiding nonsteroidal anti-inflammatory drugs due to increased bleeding risk [18,20]. This selection strategy, together with local clinical practices favoring CK testing in symptomatic patients, may partially explain the higher proportion of hospitalized patients and the observed frequency of rhabdomyolysis.

The mean age of patients in this study differed slightly from that reported in the literature on benign acute childhood myositis (BACM), which typically occurs between five and six years of age [4,24, 25, 26, 27], although still within the school-age range classically associated with the condition [9,12]. Dengue cases peak during and after the rainy season [19], and in this study, as in the literature on BACM, dengue myositis was more common during the winter, with 56.25 % of cases [4,24,25]. This differs from a prospective Brazilian study of 18 patients, which showed a more homogeneous distribution across autumn (38.9 %), spring (33.3 %), and winter (22.3 %) [22]. Rash was observed at a higher frequency in this study than the average 4 % described for BACM [12]. Although rapid serological testing may be subject to cross-reactivity in endemic regions, the diagnostic approach followed WHO recommendations, was aligned with symptom duration (NS1 early, IgM later), and incorporated clinical compatibility and exclusion criteria, supporting dengue infection as the etiological correlate of myositis in this cohort [18,20].

Based on recent definitions [14]. rhabdomyolysis was a relatively frequent complication of dengue-associated myositis in this cohort (63 %), with 46 % of patients classified as mild and 17 % as severe. This frequency should be interpreted in light of the clinically selected nature of the cohort and the CK thresholds adopted. Alternative pediatric definitions, such as CK levels exceeding five times the upper limit of normal or > 10,000 U/L for severe rhabdomyolysis, may result in lower prevalence estimates. Differences in diagnostic criteria may therefore explain the discrepancy with previous reports [11,14,21].

Nevertheless, none of the patients developed acute kidney injury. While hospitalization rates for myositis range from 4 % to 100 % in the literature [25], the higher rate (70.5 %) may be explained by overestimation of severity and the lack of standardized management protocols for dengue myositis. Pediatricians often admitted patients based solely on CK levels at presentation, even though only 17 % met criteria for severe rhabdomyolysis [22]. About 46 % of children with CK levels between 1000–5000 U/L who were hospitalized had no other risk criteria for admission. Nonetheless, the average time to symptom resolution and length of hospital stay were similar to previous studies [4,24,25].

Like other viral myositis, dengue-associated myositis demonstrated a benign and self-limiting course. Symptoms such as fever, muscle pain, and weakness may mimic other musculoskeletal conditions and should therefore be considered in the differential diagnosis. In typical cases, further investigations are unnecessary. Additional evaluation should be considered in patients with persistent muscle symptoms, neurological alterations, sustained

CK elevation, or urinary symptoms [4,24]. Severe or recurrent cases may require genetic or neurophysiological testing, based on the hypothesis that myositis may trigger or exacerbate underlying genetic conditions that predispose to metabolic impairment of skeletal muscle, such as metabolic myopathies [4,16,24].

Initial laboratory work-up should include complete blood count, CK, renal function, liver enzymes (AST and ALT), electrolytes, LDH, Erythrocyte Sedimentation Rate (ESR), venous blood gas, and urinalysis. These tests are crucial due to the risk of acute kidney injury, as the main causes of rhabdomyolysis and acute kidney injury in children include viral myositis, trauma, surgery, seizure-induced rhabdomyolysis, and physical exertion, although with lower prevalence. Pediatric patients with acute kidney injury have worse prognoses and a higher risk of progression to chronic kidney disease [11,15].

CK values between 5000–10,000 U/L, myoglobinuria, and acute kidney injury indicate severe rhabdomyolysis [14]. Serum CK peaks and myoglobinuria are associated with an increased risk of acute kidney injury, although reliable predictors for this complication in infectious myositis remain limited [11,14,24, 25, 26, 27, 28]. The risk is particularly higher when CK exceeds 5000 U/L or when myoglobinuria is accompanied by metabolic acidosis (bicarbonate < 23 mmol/L) [11]. In non-traumatic rhabdomyolysis, CK levels typically rise within 2–12 h, peak within 24–72 h, and normalize within approximately 7 days. Therefore, laboratory reassessment is recommended within 24–72 h and again after 10–15 days to confirm resolution [2,9,11,14]. In the studied cohort, although most hospitalized patients had CK levels >1000 U/L, none developed acute kidney injury, suggesting that admission decisions should not rely solely on CK values [6,11,24,25].

Urinary myoglobin testing, though often used for rhabdomyolysis screening, has low sensitivity and limited real-time availability, delaying emergency decisions [29]. Urinalysis is a reliable alternative, particularly in the absence of hematuria or when only trace amounts are present, serving as a negative predictor for myoglobinuria [15,21,30]. In this study, KDIGO criteria (urea, creatinine, and urine output monitoring) were used, making myoglobin testing unnecessary [15].

WHO guidelines for dengue include warning signs such as severe and persistent abdominal pain, persistent vomiting, fluid accumulation (ascites, pleural or pericardial effusion), postural hypotension, hepatomegaly > 2 cm below the costal margin, mucosal bleeding, lethargy or irritability. In dengue-associated myositis, these warning signs should also be regarded as *red flags* to guide clinical management, including intravenous hydration, close monitoring, and hospitalization [18,20].

Patients with atypical presentations deviating from the benign course of myositis should be evaluated for alternative diagnoses, including encephalitis, acute flaccid paralysis, Guillain-Barré syndrome, immune-mediated necrotizing myopathy, fulminant myositis, and other autoimmune neuromuscular disorders [5,12,13]. These conditions are typically characterized by progressive CK elevation, persistent muscle symptoms, and neurological abnormalities such as focal deficits, meningeal signs, altered reflexes or tone, or progressive motor weakness during hospitalization or follow-up [10].

Based on the findings of this study, the authors suggest a preliminary clinical framework, not yet validated, that stratifies patients with dengue-associated myositis into two risk groups: low risk and high risk. This stratification may serve as a potential clinical tool to support therapeutic decision-making:

• **Low-Risk:** mild symptoms, no neurological alterations, no dengue warning signs, good oral intake, no comorbidities associated with poor clinical outcomes, normal urinalysis, normal electrolytes, urea, and creatinine, adequate urine output, and CK <5000 U/L. These patients may be discharged with oral hydration, analgesics, and follow-up with clinical and laboratory reassessment within 24–72 h, a critical period for potential CPK peak and risk of acute kidney injury.
• **High-Risk:** severe refractory pain, inability to walk, neurological alterations, dengue warning signs, persistent vomiting, comorbidities associated with poor clinical outcomes, abnormal urinalysis (hematuria or proteinuria), metabolic acidosis (pH < 7.35 and HCO^-^_3_ < 23 mmol/L), elevated urea and creatinine, electrolyte disturbances, reduced urine output, or CK > 5000 U/L. These patients should be hospitalized for intravenous hydration, analgesia, rest, and serial monitoring.

This study has limitations inherent to its retrospective design, relying on information from medical records. Consequently, not all data could be collected, and the restricted number of subgroups limited the performance of multivariate analyses. Therefore, the study population represents a clinically selected subset of patients investigated for muscle involvement rather than a representative cohort of pediatric dengue cases. Despite these limitations, the study provides relevant clinical and laboratory insights into dengue-associated myositis and may help inform clinical decision-making in similar symptomatic pediatric populations.

Dengue-associated myositis in children appears to be a benign and self-limited condition, with favorable outcomes and a low need for invasive interventions. Recognition of this condition is essential in dengue-endemic regions, as its symptoms may mimic other musculoskeletal and neurological diseases. The study emphasizes that isolated CK values should not be used as the sole criterion for hospitalization (except for CK levels >5000 U/L) and that an approach based on clinical and laboratory risk stratification may help avoid unnecessary hospitalizations, reduce costs, and optimize pediatric care.

## Funding

This research received no external funding. All resources were provided by the authors.

## Authors’ contributions

The conception and design of the study: Raquel O. Birne, Marcos A. Matos, Juliana B. Goulardins.

Acquisition of data: Raquel O. Birne, Marianna A. Neri, Camille A. Rossetti, Mila O. Sena, Laís P.R. Duarte, Luji I. Takenami.

Analysis and interpretation of data: Raquel O. Birne, Marcos A. Matos, Juliana B. Goulardins, Marianna A. Neri.

Drafting the article or revising it critically for important intellectual contente: Raquel O. Birne, Marcos A. Matos, Juliana B. Goulardins.

Final approval of the version to be submitted: Raquel O. Birne, Marcos A. Matos, Juliana B. Goulardins, Marianna A. Neri, Camille A. Rossetti, Mila O. Sena, Laís P.R. Duarte, Luji I. Takenami.

## Data availability

The datasets generated and analyzed during the current study are available from the corresponding author upon reasonable request.

## Conflicts of interest

The authors declare no conflicts of interest.
